# A real-world pharmacovigilance study of romidepsin based on FDA adverse event reporting system database

**DOI:** 10.3389/fonc.2026.1694602

**Published:** 2026-06-10

**Authors:** Yuqin Yang, Yaping Huang, Yifan Wang, Yan Chen, Chengjie Ke, Maohua Chen

**Affiliations:** 1 Department of Pharmacy, The Second People's Hospital, Wuhu, Anhui, China; 2 Department of Pharmacy, Fujian Provincial Hospital, Fuzhou University Affiliated Provincial Hospital, Fuzhou, Fujian, China; 3 College of Pharmacy, China Pharmaceutical University, Nanjing, Jiangsu, China; 4 Department of Pharmacy, Pingtan Comprehensive Experimental Area Hospital, Pingtan Comprehensive Experimental Area, Fuzhou, China; 5 Department of Pharmacy, National Regional Medical Center, Binhai Campus of the First Affiliated Hospital, Fujian Medical University, Fuzhou, China; 6 Department of Pharmacy, The First Affiliated Hospital, Fujian Medical University, Fuzhou, China

**Keywords:** cutaneous T-cell lymphomas, FAERS, peripheral T-cell lymphomas, pharmacovigilance study, romidepsin

## Abstract

**Background:**

Romidepsin is a class I-selective histone deacetylase (HDAC) inhibitor indicated for the treatment of adult patients with cutaneous T-cell lymphoma (CTCL) who have received at least one prior systemic therapy and for adult patients with relapsed or refractory peripheral T-cell lymphoma (PTCL). Despite its clinical use, large-scale safety data remain limited. This study leverages the FDA Adverse Event Reporting System (FAERS) database to characterize romidepsin-associated adverse events (AEs) in real-world settings, aiming to enhance clinical risk management.

**Methods:**

Data from the FAERS database, spanning from January 2014 to March 2025, served as the basis for this analysis. To evaluate the association between romidepsin and AEs, multiple disproportionality analyses were employed, including the reporting odds ratio (ROR), the proportional reporting ratio (PRR), the Bayesian confidence propagation neural network (BCPNN), and the multi-item gamma Poisson shrinker (MGPS).

**Results:**

Within the specified reporting period, 17,448,626 AE reports were recorded in the FAERS database, of which 1,285 events were associated with romidepsin. Based on four calculation methods, 101 preferred terms (PTs) related to romidepsin were determined. Common AEs included thrombocytopenia, pyrexia, anemia, electrocardiogram QT prolonged, and infections, aligning with the drug label. In addition, some unanticipated major AEs were detected, including cardiotoxicities beyond QT prolongation (acute cardiac failure, atrial fibrillation, sinus tachycardia, mitral valve incompetence, and bundle branch block left) alongside other significant AEs: hepatic failure, amenorrhea, mental status changes, glomerular filtration rate decreased, embolism, and retinal detachment.

**Conclusion:**

This study provides a systematic evaluation of AEs associated with romidepsin, confirming its established safety profile and identifying several emerging safety concerns in a real-world setting. These findings offer valuable insights to assist clinicians and pharmacists in better-managing romidepsin’s safety.

## 1 Introduction

T-cell lymphomas (TCLs) represent a rare, heterogeneous, and often aggressive subset of non-Hodgkin’s lymphomas (NHLs) (1, 2). Clinically, they are divided into peripheral T-cell lymphomas (PTCLs) and cutaneous T-cell lymphomas (CTCLs), each reflecting distinct biological characteristics and disease manifestations. PTCLs are characterized by their high aggressiveness, poor differentiation, and unfavorable clinical outcomes. Current therapeutic strategies for PTCL remain limited, with few effective treatment options available. While ALK-positive anaplastic large cell lymphoma (ALCL) demonstrates significant responses to CHOP (cyclophosphamide, doxorubicin, vincristine, and prednisone)-based regimens or CHP in combination with brentuximab vedotin, other PTCL subtypes show suboptimal remission rates and dismal prognoses following first-line therapies. Furthermore, PTCL is often associated with a high rate of relapse; approximately 70% of patients develop relapsed/refractory (R/R) disease (3), with these patients experiencing a median overall survival (OS) ranging from 6 to 12 months (4–6).

Romidepsin, a class I-selective histone deacetylase (HDAC) inhibitor, exerts antitumor effects through multiple mechanisms, including the induction of apoptosis, suppression of angiogenesis, and cell cycle arrest (3). It has demonstrated significant clinical efficacy in TCLs. In clinical trials assessing romidepsin in CTCL, two studies involving 71 and 96 patients reported response rates of 34% and 38%, with median durations of response (DOR) of 13.7 and 15 months, respectively (7, 8). These results facilitated the FDA’s approval of romidepsin for the treatment of CTCL in adult patients who had received at least one prior systemic therapy in 2009. Subsequently, two Phase II trials targeting PTCL enrolled 47 and 131 patients, demonstrating response rates of 38% and 25%, with median DOR of 8.9 and 17 months, respectively (9, 10). These promising outcomes led to the FDA’s accelerated approval of romidepsin in 2011 for the treatment of relapsed/refractory (R/R) PTCL in patients with ≥1 prior systemic therapy. Despite the failure of a confirmatory Phase III trial (N = 421) to meet its primary endpoint of improved progression-free survival (PFS) for romidepsin plus CHOP (Ro-CHOP) compared to CHOP alone in previously untreated PTCL patients (11), the NCCN Guidelines for PTCL continue to recommend romidepsin as a key therapeutic option for R/R PTCL (12), based on earlier Phase II data and subsequent studies (13, 14). An exploratory analysis of the same Phase III cohort, following a 6-year follow-up, revealed a potential benefit specifically in the follicular helper T-cell lymphoma subgroup, where median PFS was 19.5 months with Ro-CHOP versus 10.6 months with CHOP alone (P = 0.039). However, no significant difference in OS was observed (P = 0.342) (15). Recent clinical findings suggest that the combination of romidepsin and duvelisib enhances efficacy while alleviating PI3K inhibitor-induced toxicity in patients with R/R TCLs (16). As a result, this romidepsin-duvelisib regimen has been incorporated into the NCCN Guidelines for second-line and subsequent therapy settings in PTCL treatment (12).

Clinical trials of romidepsin have identified nausea, fatigue, infections, vomiting, anorexia, anemia, thrombocytopenia, neutropenia, lymphopenia, and electrocardiographic ST-T wave changes as the most commonly reported adverse events (AEs) (7–10). Infections, fatigue, dyspnea, QT prolongation, and hypomagnesemia were among the most frequent AEs leading to treatment discontinuation (17). Current understanding of romidepsin-associated AEs is primarily based on clinical trial data, with limited contributions from real-world evidence. The stringent eligibility criteria and small sample sizes inherent in these trials may fail to fully capture the drug’s safety profile in broader, more heterogeneous populations. As such, the spectrum of AEs encountered in real-world settings may exceed those reported in controlled clinical studies.

The FDA Adverse Event Reporting System (FAERS), a comprehensive repository for post-marketing pharmacovigilance, facilitates systematic risk–benefit assessments by capturing real-world AEs (18). In contrast to clinical trials, FAERS data encompass a broad spectrum of patient experiences, including rare or delayed-onset AEs that may elude detection in time-constrained studies. Continuous surveillance via FAERS is therefore essential for rigorous post-marketing safety evaluation, particularly as the clinical application of romidepsin continues to expand. Although romidepsin represents a promising therapeutic agent for T-cell lymphomas, its safety profile warrants further elucidation through real-world data. This study aims to investigate post-marketing AEs associated with romidepsin using FAERS data, thereby generating evidence to inform and enhance its clinical safety management. Our study is compliant with the TITAN Guidelines 2025 - governing declaration and use of AI (19).

## 2 Methods

### 2.1 Study design and data source

This study conducted a pharmacovigilance analysis using the FAERS database to investigate the association between romidepsin and AEs. The method calculated the proportion of romidepsin-associated AEs relative to all other drug-related AEs in the FAERS database. A statistically significant signal was defined as a disproportionate increase in AE reporting frequency for romidepsin compared to other medications (20).

This study complied with FDA protocols for data extraction from the FAERS database (source: https://fis.fda.gov/extensions/FPD-QDE-FAERS/FPD-QDE-FAERS.html). To comprehensively evaluate romidepsin’s post-marketing safety profile, reports spanning January 2014 through March 2025 were analyzed.

### 2.2 Data extraction

In compliance with FDA data governance protocols, this study implemented multi-level deduplication strategies to refine the raw dataset, effectively eliminating duplicate entries and enhancing data reliability (20–22). The deduplication process was conducted based on a combination of the patient ID (CASEID) and the FDA receipt date (FDA_DT). For reports with an identical CASEID, the record with the most recent FDA_DT was retained. If both the CASEID and the FDA_DT were identical, a manual review was performed using additional attributes, such as the primary suspect drug, the event outcome, and the reporter type, in order to retain the report that was both the most complete and the most clinically relevant (23, 24). This multidimensional approach ensured the integrity of the final analytical dataset (25). A targeted search algorithm (“ROMIDEPSIN” OR “ISODAX”) combined with primary suspect (PS) designation was systematically employed to identify romidepsin-associated AE reports (26). All AE terms in the raw data were standardized using the Medical Dictionary for Regulatory Activities (MedDRA) version 26.0. A Python script was used to automatically map the terms in the reports to their corresponding Preferred Terms (PTs) and System Organ Classes (SOCs) (27). To ensure coding consistency, dual independent coding and cross-validation were performed for high-frequency and unexpected AEs. In cases of coding discrepancies, a third senior researcher (the corresponding author) made the final decision to guarantee the accuracy and consistency of the coding (27). Data processing was carried out using Python 3.10 (Python Software Foundation, Holland), Microsoft Excel 2019, and GraphPad Prism 8 (GraphPad Software, CA, USA), ensuring a systematic and precise approach to handling the dataset.

**Figure 1:**
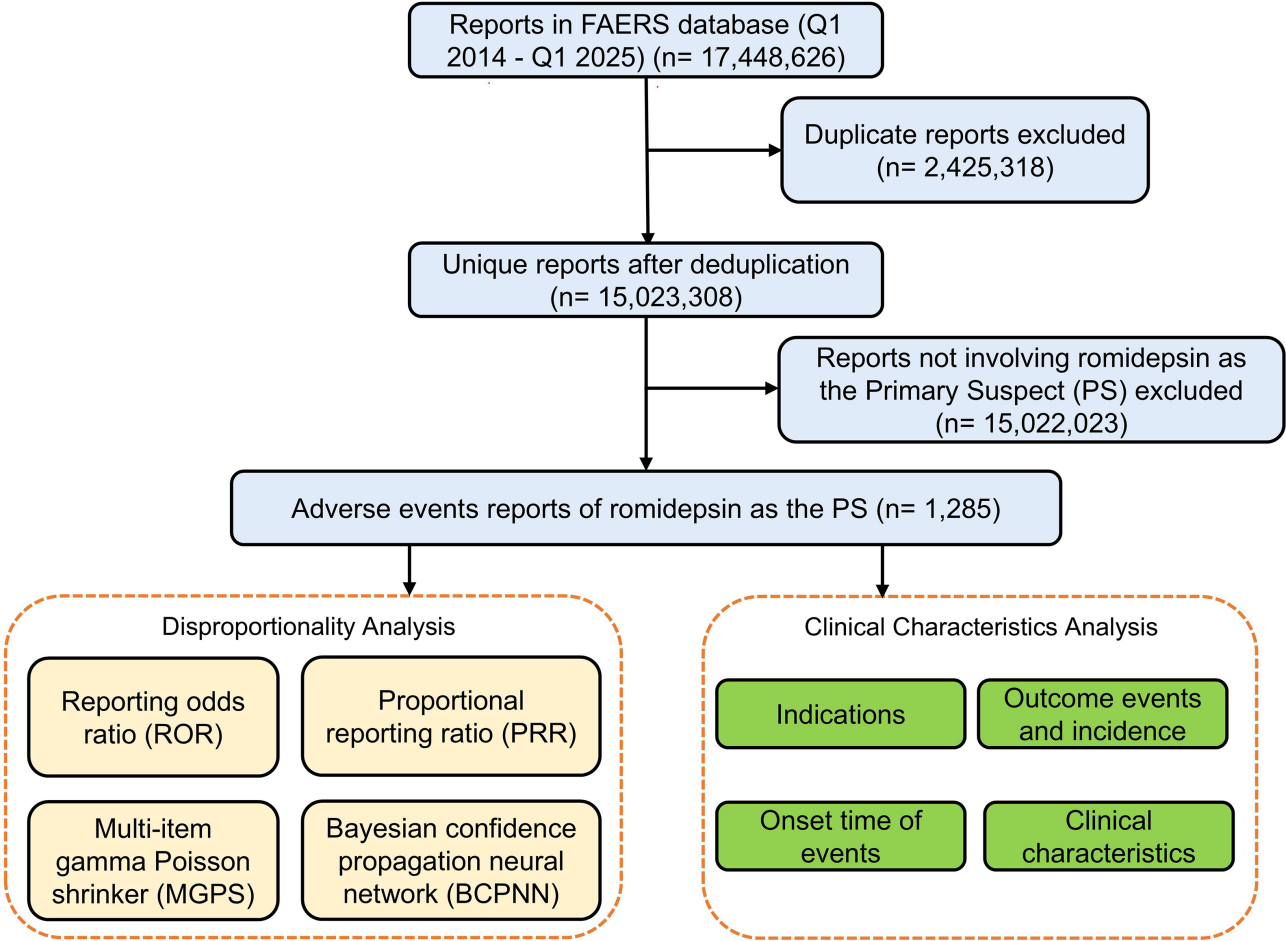
The flow diagram of selecting romidepsin-related AEs from the FAERS database.

This romidepsin pharmacovigilance study employed multifaceted characterization, systematically evaluating critical AE aspects including salient patient characteristics (age, weight), manifestations of severe outcomes, geographical case distribution, therapeutic indications, and reporter roles. The analysis concluded with the creation of an intuitive flowchart presenting the results (**Figure 1**).

### 2.3 Data analysis

This study operationalized quantitative signal detection measures derived from 2×2 frequency matrices (**Supplementary Table 1**), adopting a multi-method assessment protocol that synergized four computational architectures: reporting odds ratio (ROR), proportional reporting ratio (PRR), Bayesian confidence propagation neural network (BCPNN), and multi-item gamma Poisson shrinker (MGPS). The integrated framework leveraged hybrid analytical paradigms combining disproportionality metrics with empirical Bayesian shrinkage estimators to quantify drug-AE correlations (20, 21, 28, 29). The specific parameter settings and signal detection thresholds for each algorithm are defined as follows. A positive ROR signal was defined as a lower limit of the 95% confidence interval (CI) exceeding 1, with at least two reports (n ≥ 2). For PRR, a signal was identified when PRR ≥ 2, χ² ≥ 4, and n ≥ 3. BCPNN generated a positive signal when the lower limit of the information component (IC025) exceeded 0. For MGPS, a signal was defined as the lower limit of the empirical Bayesian geometric mean (EBGM05) exceeding 2, regardless of the number of reports (n ≥ 0). Detailed computational architectures and validation thresholds for each algorithm are summarized in **Supplementary Table 2**, which provides granular specifications for each methodological implementation.

## 3 Results

### 3.1 Characteristics of AE reports

The FAERS database documented 17,448,626 AE reports between January 2014 and March 2025. Following deduplication, 1,285 cases were specifically associated with romidepsin. The clinical profiles of these patients are summarized in **Table 1**. Gender distribution showed that males comprised a marginally higher representation at 55.67% of the cohort. Patients predominantly fell within the 18–65 age bracket (50.65%), followed by those > 65 years (48.38%), with pediatric cases (< 18 years) representing 0.97%. The median age at AE occurrence was 65 years (interquartile range (IQR): 54-74). Weight distribution indicated that 71.07% of patients weighed < 80 kg, with a median weight of 68 kg (IQR: 57-81). Geographically, the United States contributed the highest proportion of reports (41.44%), followed by Japan (20.80%) and France (12.20%). The primary therapeutic indication was unspecified PTCLs (24.76%), with angioimmunoblastic TCLs (15.58%) and CTCLs (11.84%) being other common diagnoses. Concomitant medications were documented in 66.85% of cases, predominantly doxorubicin (25.73%), cyclophosphamide (23.52%), and vincristine (19.09%). Serious outcomes occurred in 89.88% of cases, with hospitalization being the most frequent (40.35%). Other significant outcomes included death (30.91%), life-threatening events (7.36%), disability (1.21%), and other severe complications (54.29%). Onset timing was specified in 21.48% of reports, demonstrating a median latency of 21.5 days (IQR: 3-69.75 days). Healthcare professionals submitted 95.77% of reports, with consumers accounting for the remaining 4.23%.

**Table 1:** Clinical characteristics of reports with romidepsin from the FAERS database (Jan. 2014 to Mar. 2025).

Characteristics	Romidepsin-induced AE reports (n = 1,285)		
Number of events	Available number, n	Case number, n	Case proportion, %
Gender, n (%)	1076	-	83.74%
Female	–	477	44.33%
Male	–	599	55.67%
Age (years), n (%)	928	-	72.22%
< 18	–	9	0.97%
18 ≤ and ≤ 65	–	470	50.65%
> 65	–	449	48.38%
Median (IQR)	–	65 (54 - 74)	–
Weight (kg), n (%)	477	-	37.12%
< 80	–	339	71.07%
80 ≤ and ≤ 100	–	97	20.34%
> 100	–	41	8.60%
Median (IQR)	–	68 (57 - 81)	–
Reported countries, n (%)	1279	-	99.53%
US	–	530	41.44%
JP	–	266	20.80%
FR	–	156	12.20%
Other country	–	327	25.57%
Indications, n (%)	1123	-	87.39%
Peripheral T-Cell Lymphoma Unspecified	–	278	24.76%
Angioimmunoblastic T-Cell Lymphoma	–	175	15.58%
Cutaneous T-Cell Lymphoma	–	133	11.84%
Combination drugs, n (%)	859	-	66.85%
Doxorubicin	–	221	25.73%
Cyclophosphamide	–	202	23.52%
Vincristine	–	164	19.09%
Outcomes, n (%)	1285	-	100.00%
Non-serious Outcome	-	130	10.12%
Serious Outcome	-	1155	89.88%
Death	–	357	30.91%
Life-threatening	–	85	7.36%
Hospitalization	–	466	40.35%
Disability	–	14	1.21%
Other serious outcomes	–	627	54.29%
Time-to-onset (days)	276	-	21.48%
Median (IQR)	–	21.5 (3-69.75)	–
Reporters, n (%)	1278	-	99.46%
Health professional	–	1224	95.77%
Consumer	–	54	4.23%
Reporting year, n (%)	1285	-	100.00%
2014	–	66	5.14%
2015	–	116	9.03%
2016	–	115	8.95%
2017	–	78	6.07%
2018	–	109	8.48%
2019	–	173	13.46%
2020	–	120	9.34%
2021	–	118	9.18%
2022	–	164	12.76%
2023	–	76	5.91%
2024	–	106	8.25%
2025	–	44	3.42%

AE, adverse event; FAERS, FDA Adverse Event Reporting System; IQR, interquartile range.

### 3.2 Disproportionality analysis

The SOC level safety signals of romidepsin are summarized in **Table 2**. Disproportionality analysis using four algorithms revealed statistically significant signals across 25 organ systems. The strongest signals were observed for blood and lymphatic system disorders and neoplasms benign, malignant and unspecified (incl cysts and polyps), followed by cardiac disorders and hepatobiliary disorders, which achieved significance in three detection methods. Additional signals with two-algorithm concordance were observed for infections and infestations, metabolism and nutrition disorders, and investigations.

**Table 2:** The signal strength of reports of romidepsin at the SOC level in the FAERS database.

System organ class (SOC)	Number	ROR (95% CI)	PRR (χ2)	IC (IC025)	EBGM (EBGM05)
Blood and lymphatic system disorders	239	5.34 (4.68-6.10)^a^	4.95 (767.40)^a^	2.31 (2.02)^a^	4.95 (4.33)^a^
Neoplasms benign, malignant and unspecified (incl cysts and polyps)	332	4.27 (3.80-4.79)^a^	3.86 (726.08)^a^	1.95 (1.74)^a^	3.86 (3.44)^a^
Cardiac disorders	142	2.31 (1.95-2.74)^a^	2.24 (99.86)^a^	1.16 (0.98)^a^	2.24 (1.89)
Hepatobiliary disorders	52	2.10 (1.60-2.76)^a^	2.08 (29.35)^a^	1.06 (0.80)^a^	2.08 (1.58)
Infections and infestations	243	1.73 (1.51-1.97)^a^	1.66 (67.23)	0.73 (0.64)^a^	1.66 (1.45)
Metabolism and nutrition disorders	102	1.63 (1.34-1.99)^a^	1.61 (24.07)	0.69 (0.56)^a^	1.61 (1.32)
Investigations	229	1.63 (1.42-1.86)^a^	1.57 (50.64)	0.65 (0.57)^a^	1.57 (1.37)
Gastrointestinal disorders	196	1.03 (0.89-1.20)	1.03 (0.19)	0.04 (0.04)^a^	1.03 (0.89)
Respiratory, thoracic and mediastinal disorders	109	0.94 (0.78-1.14)	0.94 (0.41)	-0.09 (-0.10)	0.94 (0.78)
General disorders and administration site conditions	444	0.94 (0.85-1.04)	0.95 (1.54)	-0.08 (-0.09)	0.95 (0.86)
Immune system disorders	38	0.92 (0.67-1.27)	0.93 (0.23)	-0.11 (-0.15)	0.93 (0.67)
Renal and urinary disorders	41	0.78 (0.57-1.06)	0.78 (2.47)	-0.35 (-0.48)	0.78 (0.58)
Vascular disorders	45	0.69 (0.51-0.92)	0.69 (6.30)	-0.53 (-0.71)	0.69 (0.52)
Ear and labyrinth disorders	9	0.64 (0.33-1.22)	0.64 (1.88)	-0.65 (-1.25)	0.64 (0.33)
Nervous system disorders	108	0.52 (0.43-0.63)	0.54 (45.91)	-0.89 (-1.08)	0.54 (0.45)
Skin and subcutaneous tissue disorders	67	0.47 (0.37-0.61)	0.49 (37.90)	-1.03 (-1.32)	0.49 (0.38)
Injury, poisoning and procedural complications	150	0.44 (0.37-0.52)	0.47 (102.69)	-1.09 (-1.29)	0.47 (0.40)
Reproductive system and breast disorders	9	0.40 (0.21-0.77)	0.40 (8.17)	-1.32 (-2.54)	0.40 (0.21)
Eye disorders	19	0.38 (0.24-0.60)	0.39 (18.90)	-1.37 (-2.16)	0.39 (0.25)
Musculoskeletal and connective tissue disorders	33	0.27 (0.19-0.38)	0.28 (64.84)	-1.85 (-2.60)	0.28 (0.20)
Endocrine disorders	2	0.24 (0.06-0.94)	0.24 (4.95)	-2.08 (-8.33)	0.24 (0.06)
Psychiatric disorders	22	0.17 (0.11-0.26)	0.18 (86.50)	-2.48 (-3.77)	0.18 (0.12)
Social circumstances	2	0.13 (0.03-0.54)	0.13 (11.20)	-2.89 (-11.58)	0.13 (0.03)
Surgical and medical procedures	4	0.08 (0.03-0.22)	0.08 (40.36)	-3.56 (-9.50)	0.08 (0.03)
Product issues	3	0.05 (0.02-0.16)	0.05 (51.39)	-4.23 (-13.11)	0.05 (0.02)

FAERS, FDA Adverse Event Reporting System; SOC, system organ class; ROR, reporting odds ratio; CI, confidence interval; PRR, proportional reporting ratio; χ^2^, chi-squared; IC, information component; IC025, the lower limit of 95% CI of the IC; EBGM, empirical Bayesian geometric mean; EBGM05, the lower limit of 95% CI of EBGM.

^a^ Indicates statistically significant signals in the algorithm.

Based on the FAERS database analysis, 101 PTs associated with romidepsin were identified, all meeting the minimum reporting threshold of ≥ 3 cases. After excluding non-drug-related AE signals potentially attributable to tumor progression, various injuries, poisoning complications, procedural complications, product issues, and surgical/medical procedures, a total of 71 PTs were confirmed as romidepsin-associated signals (**Table 3**). Non-drug-related AE signals potentially caused by disease progression or other factors are detailed in **Supplementary Table 3**. The top 10 PTs by case count and by ROR are presented in **Supplementary Tables 4**, **5**, respectively. In addition to the known AEs already listed by the FDA, this study identified several AEs that had not previously been documented and that are significantly associated with romidepsin. These newly identified AEs are shown in bold in **Table 3**.

**Table 3:** The signal strength of reports of romidepsin at PT level in the FAERS database (n ≥ 3).

Preferred terms (PTs)	Number	ROR (95% CI)	PRR (χ^2^)	IC (IC025)	EBGM (EBGM05)
Thrombocytopenia	90	16.00 (12.98-19.73)	15.61 (1231.29)	3.96 (3.21)	15.59 (12.65)
Pyrexia	80	4.48 (3.59-5.59)	4.40 (210.93)	2.14 (1.71)	4.39 (3.52)
Anaemia	63	6.44 (5.02-8.26)	6.34 (283.96)	2.66 (2.08)	6.34 (4.94)
Electrocardiogram QT prolonged	62	31.70 (24.65-40.77)	31.15 (1805.87)	4.96 (3.86)	31.08 (24.17)
Platelet count decreased	61	10.62 (8.24-13.68)	10.45 (521.66)	3.38 (2.63)	10.44 (8.10)
**Atrial fibrillation**	56	10.99 (8.44-14.31)	10.83 (499.84)	3.44 (2.64)	10.82 (8.31)
Neutropenia	50	6.52 (4.93-8.62)	6.44 (230.10)	2.69 (2.03)	6.44 (4.87)
Decreased appetite	41	3.13 (2.30-4.26)	3.10 (58.70)	1.63 (1.20)	3.10 (2.28)
Febrile neutropenia	38	10.56 (7.67-14.54)	10.45 (324.87)	3.38 (2.46)	10.44 (7.58)
Sepsis	29	4.97 (3.45-7.16)	4.93 (91.05)	2.30 (1.60)	4.93 (3.42)
White blood cell count decreased	25	4.12 (2.78-6.11)	4.10 (58.63)	2.03 (1.37)	4.10 (2.76)
Neutrophil count decreased	23	10.32 (6.85-15.56)	10.26 (192.16)	3.36 (2.23)	10.25 (6.80)
Tumour lysis syndrome	20	41.02 (26.41-63.70)	40.78 (773.82)	5.35 (3.44)	40.66 (26.18)
Pancytopenia	19	6.77 (4.31-10.63)	6.74 (92.91)	2.75 (1.75)	6.74 (4.29)
Respiratory failure	16	4.47 (2.73-7.30)	4.45 (42.87)	2.15 (1.32)	4.45 (2.72)
Cytopenia	16	20.65 (12.63-33.75)	20.56 (297.25)	4.36 (2.67)	20.52 (12.55)
Dysgeusia	14	3.68 (2.18-6.23)	3.67 (27.25)	1.88 (1.11)	3.67 (2.17)
**Mouth ulceration**	14	12.67 (7.49-21.42)	12.62 (149.72)	3.66 (2.16)	12.61 (7.46)
Liver disorder	14	6.23 (3.68-10.53)	6.21 (61.15)	2.63 (1.56)	6.20 (3.67)
Lymphocyte count decreased	13	11.82 (6.85-20.38)	11.77 (128.10)	3.56 (2.06)	11.76 (6.82)
**Hepatic failure**	11	7.83 (4.33-14.16)	7.81 (65.34)	2.97 (1.64)	7.81 (4.32)
Septic shock	10	4.39 (2.36-8.17)	4.38 (26.09)	2.13 (1.15)	4.38 (2.35)
Leukopenia	10	3.89 (2.09-7.23)	3.88 (21.39)	1.96 (1.05)	3.88 (2.09)
Haematotoxicity	10	19.54 (10.50-36.36)	19.48 (175.10)	4.28 (2.30)	19.45 (10.45)
Myelosuppression	10	5.40 (2.90-10.04)	5.38 (35.69)	2.43 (1.30)	5.38 (2.89)
Cytomegalovirus infection	9	9.87 (5.13-18.98)	9.84 (71.47)	3.30 (1.71)	9.84 (5.11)
**Amenorrhoea**	9	12.42 (6.46-23.90)	12.39 (94.19)	3.63 (1.89)	12.38 (6.44)
Taste disorder	9	6.96 (3.62-13.39)	6.94 (45.78)	2.80 (1.45)	6.94 (3.61)
Hepatic function abnormal	8	4.28 (2.14-8.57)	4.28 (20.08)	2.10 (1.05)	4.28 (2.14)
Epstein-Barr virus infection	7	20.85 (9.93-43.80)	20.81 (131.80)	4.38 (2.08)	20.78 (9.89)
Lymphopenia	7	8.75 (4.17-18.38)	8.74 (47.94)	3.13 (1.49)	8.73 (4.16)
Clostridium difficile colitis	7	12.45 (5.93-26.14)	12.43 (73.48)	3.63 (1.73)	12.41 (5.91)
**Sinus tachycardia**	7	10.84 (5.16-22.75)	10.82 (62.32)	3.43 (1.64)	10.81 (5.15)
Blood lactate dehydrogenase increased	7	10.56 (5.03-22.17)	10.54 (60.40)	3.40 (1.62)	10.53 (5.02)
Electrocardiogram abnormal	6	16.35 (7.34-36.43)	16.32 (86.19)	4.03 (1.81)	16.30 (7.31)
**Mental status changes**	6	4.86 (2.18-10.82)	4.85 (18.34)	2.28 (1.02)	4.85 (2.18)
Urosepsis	6	12.11 (5.43-26.99)	12.09 (60.99)	3.59 (1.61)	12.08 (5.42)
Bone marrow failure	6	5.39 (2.42-12.01)	5.38 (21.40)	2.43 (1.09)	5.38 (2.41)
Cytomegalovirus viraemia	6	23.87 (10.71-53.22)	23.83 (131.01)	4.57 (2.05)	23.79 (10.67)
Lung infiltration	6	18.45 (8.28-41.12)	18.42 (98.71)	4.20 (1.88)	18.39 (8.25)
Epstein-Barr virus infection reactivation	6	80.46 (36.03-179.68)	80.32 (467.09)	6.32 (2.83)	79.83 (35.75)
Cytomegalovirus infection reactivation	6	23.59 (10.58-52.59)	23.55 (129.35)	4.56 (2.04)	23.51 (10.55)
Electrolyte imbalance	5	8.46 (3.52-20.34)	8.45 (32.82)	3.08 (1.28)	8.44 (3.51)
Acute pulmonary oedema	5	19.83 (8.24-47.70)	19.80 (89.12)	4.31 (1.79)	19.77 (8.22)
Cardiotoxicity	5	9.79 (4.07-23.55)	9.78 (39.39)	3.29 (1.37)	9.77 (4.06)
Wound infection	5	8.58 (3.57-20.63)	8.57 (33.40)	3.10 (1.29)	8.56 (3.56)
Febrile bone marrow aplasia	5	21.86 (9.08-52.58)	21.82 (99.19)	4.45 (1.85)	21.79 (9.06)
Orthostatic hypotension	5	5.55 (2.31-13.33)	5.54 (18.59)	2.47 (1.03)	5.54 (2.30)
Cytomegalovirus chorioretinitis	4	37.27 (13.96-99.51)	37.23 (140.63)	5.21 (1.95)	37.13 (13.91)
Pyoderma gangrenosum	4	19.74 (7.40-52.67)	19.72 (70.98)	4.30 (1.61)	19.69 (7.38)
**Cardiac failure acute**	4	10.71 (4.01-28.56)	10.70 (35.13)	3.42 (1.28)	10.69 (4.01)
**Extravasation**	4	20.25 (7.59-54.02)	20.23 (72.99)	4.34 (1.63)	20.20 (7.57)
Staphylococcal sepsis	4	18.34 (6.87-48.92)	18.32 (65.39)	4.19 (1.57)	18.29 (6.86)
**Glomerular filtration rate decreased**	4	5.72 (2.14-15.24)	5.71 (15.54)	2.51 (0.94)	5.71 (2.14)
Folliculitis	4	6.61 (2.48-17.63)	6.60 (19.01)	2.72 (1.02)	6.60 (2.48)
Escherichia sepsis	3	20.64 (6.65-64.08)	20.62 (55.92)	4.36 (1.41)	20.59 (6.63)
**Embolism**	3	7.35 (2.37-22.81)	7.34 (16.43)	2.88 (0.93)	7.34 (2.37)
**Retinal detachment**	3	6.37 (2.05-19.77)	6.37 (13.56)	2.67 (0.86)	6.36 (2.05)
Infusion site extravasation	3	7.80 (2.51-24.19)	7.79 (17.74)	2.96 (0.95)	7.79 (2.51)
Gastrointestinal sounds abnormal	3	11.67 (3.76-36.22)	11.66 (29.21)	3.54 (1.14)	11.65 (3.75)
Parosmia	3	7.90 (2.55-24.52)	7.90 (18.06)	2.98 (0.96)	7.89 (2.54)
Localised oedema	3	12.28 (3.96-38.13)	12.27 (31.04)	3.62 (1.17)	12.26 (3.95)
Hypoalbuminaemia	3	8.04 (2.59-24.95)	8.03 (18.46)	3.01 (0.97)	8.03 (2.59)
**Mitral valve incompetence**	3	6.85 (2.21-21.26)	6.85 (14.97)	2.77 (0.89)	6.84 (2.21)
**Bundle branch block left**	3	16.24 (5.23-50.42)	16.23 (42.82)	4.02 (1.29)	16.21 (5.22)
Pneumonia klebsiella	3	30.33 (9.76-94.21)	30.30 (84.81)	4.92 (1.58)	30.23 (9.73)
Cystitis haemorrhagic	3	13.69 (4.41-42.49)	13.68 (35.22)	3.77 (1.22)	13.66 (4.40)
Gastrointestinal toxicity	3	11.55 (3.72-35.84)	11.54 (28.85)	3.53 (1.14)	11.53 (3.71)
Platelet count abnormal	3	8.16 (2.63-25.31)	8.15 (18.81)	3.03 (0.98)	8.15 (2.62)
Haemoglobin abnormal	3	7.93 (2.55-24.60)	7.92 (18.13)	2.98 (0.96)	7.92 (2.55)
Staphylococcal bacteraemia	3	13.78 (4.44-42.77)	13.77 (35.48)	3.78 (1.22)	13.75 (4.43)

FAERS, FDA Adverse Event Reporting System; ROR, reporting odds ratio; CI, confidence interval; PRR, proportional reporting ratio; χ^2^, chi-squared; IC, information component; EBGM, empirical Bayesian geometric mean.

The bold data indicate AEs associated with romidepsin that were not previously documented.

### 3.3 Onset time of AEs

**Figure 2:**
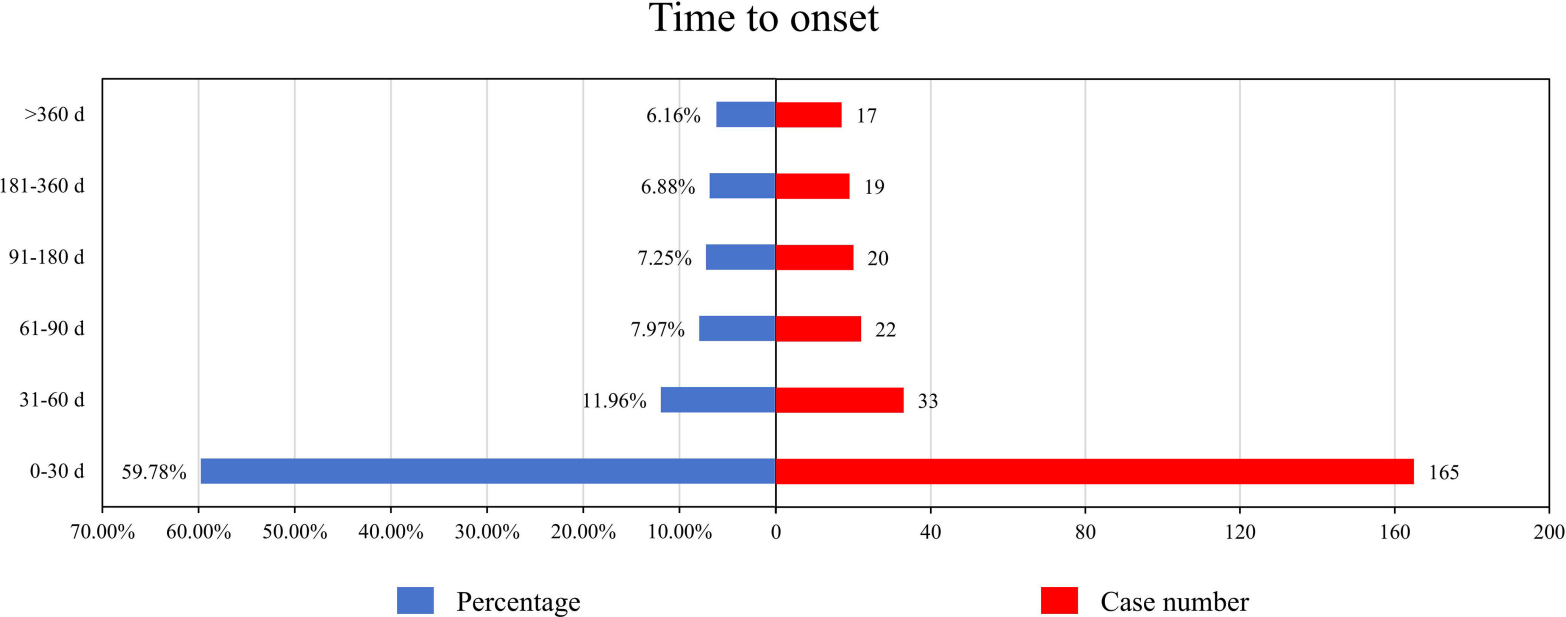
Time-to-onset of romidepsin-related AEs at the overall level.

Temporal distribution analysis of romidepsin-related AEs identified 276 reports (21.48% of total cases) with verifiable onset data after excluding entries lacking temporal specificity. The median latency period was 21.5 days (interquartile range: 3-69.75 days). Approximately 59.78% (165/276) of patients experienced AEs within the first month after the initiation of romidepsin treatment. Furthermore, the proportion of patients with AEs in the second month (n = 33, 11.96%) and the third month (n = 22, 7.97%) was significantly lower than that in the first month (**Figure 2**), highlighting the critical importance of early monitoring for romidepsin-associated AEs.

## 4 Discussion

This study utilized the extensive FAERS database to investigate the potential association between romidepsin and AEs, evaluating its post-marketing safety profile. This analysis revealed that among reported AEs, males constituted a higher proportion of cases (55.67%) compared to females (44.33%), with a median patient age of 65 years (IQR: 54–74). These findings underscore the need for vigilant AE monitoring in elderly male patients, who demonstrated a higher reporting frequency. This demographic profile aligns with the established epidemiology of TCLs, which show higher incidence in males and increasing frequency with age, although certain PTCL subtypes, such as ALK-positive anaplastic large cell lymphoma (ALK+ALCL) and hepatosplenic T-cell lymphoma (HSTL), are more prevalent in younger populations (2, 30, 31). Recent global cancer data indicate that NHL incidence rates are approximately twice as high in developed nations compared to developing nations, with the highest rates observed in North America (30). Our analysis reveals that geographically, the majority of AE reports originated from the United States (41.44%) and Japan (20.80%). This distribution pattern may be partially attributed to the higher documented incidence of NHL, including TCLs, within these countries. The pharmacovigilance analysis indicated a rapid onset of many toxicities. Notably, 59.78% of romidepsin-associated AEs occurred within the first 30 days of treatment, aligning with prior observations (10, 14). Quantitative analysis confirmed this pattern, demonstrating a median time-to-onset of 21.5 days (IQR: 3-69.75). These temporal characteristics underscore the need for heightened clinical vigilance during the initial treatment phase.

The analysis demonstrates that significant AEs associated with romidepsin primarily affect several SOCs, including blood and lymphatic system disorders, cardiac disorders, hepatobiliary disorders, infections and infestations. The most frequently associated PTs were thrombocytopenia, pyrexia, anemia, electrocardiogram QT prolonged, and infections. These results are consistent with prior safety studies of romidepsin, confirming established knowledge of its safety profile. Furthermore, this study identified previously undocumented AEs in the reference core prescribing information (RCP), including acute cardiac failure, hepatic failure, amenorrhea, mental status change, etc. These findings enhance the clinical understanding of romidepsin’s risk profile and may inform optimized pharmacovigilance strategies and risk management protocols.

Hematological toxicities represent the most frequent AEs associated with romidepsin therapy. These commonly include thrombocytopenia, neutropenia, white blood cell count decreased, anemia, and lymphocyte count decreased. Our pharmacovigilance findings confirm that, within blood and lymphatic system disorders, thrombocytopenia was the most prevalent AE signal (n = 90). This was followed by anemia (n = 63), neutropenia (n = 50), febrile neutropenia (n = 38), white blood cell count decreased (n = 25). A comprehensive meta-analysis of romidepsin trials indicates that lymphopenia and neutropenia are the predominant grade ≥ 3 AEs, occurring in 46% and 28% of patients, respectively (32). As demonstrated by Coiffier et al. (9), single-agent romidepsin in patients with R/R PTCL resulted in grade ≥ 3 thrombocytopenia in 23% of patients and grade ≥ 3 neutropenia in 18%. Thrombocytopenia was a leading cause of romidepsin dose modifications (interruptions or reductions). Given the high risk of hematological toxicity with romidepsin-containing regimens, regular blood count monitoring (typically 1–2 times per week) should be considered standard practice. This monitoring is essential to detect toxicities early and guide potential therapy adjustments based on clinical need.

It is worth noting that infection has a significant incidence rate among patients with PTCL. Registry analyses have identified it as the second leading cause of death after disease progression (33). Treatment with HDAC inhibitors, including romidepsin, has been associated with infectious complications in PTCL patients (34). Our study confirmed infections and infestations as a significant signal at the SOC level (ROR 1.73). Key infection-related AEs specifically signaled for romidepsin included sepsis (ROR 4.97), septic shock (ROR 4.39), cytomegalovirus infection (ROR 9.87), Epstein-Barr virus (EBV) infection (ROR 20.85), clostridium difficile colitis (ROR 12.45), and urosepsis (ROR 12.11). A phase II trial observed infections in 36% (17/47) patients over 28 cycles, with bacterial infections being the most frequent, followed by fungal and viral infections (10). Another phase II study in R/R PTCL reported all-grade infections related to romidepsin monotherapy in approximately 18% (24/131) of patients, with grade ≥ 3 infections occurring in about 6% (8/131). Infection-related complications led to death in 3.8% (5/131) of patients (9). A meta-analysis of romidepsin studies in PTCL found an overall all-grade infection rate of 12.8%, with grade ≥ 3 infection-related AEs occurring in 5% of cases (32). Given that infectious complications contribute substantially to mortality, clinicians should maintain vigilance, actively monitor patients receiving romidepsin for infections, and implement antimicrobial prophylaxis according to clinical guidelines. Furthermore, HDAC inhibitors may induce severe EBV reactivation in patients with extranodal NK/T-cell lymphoma (ENKTL), a specific PTCL subtype (35–37). The underlying mechanism involves HDAC inhibitor-mediated epigenetic modulation of the EBV genome. During latency, EBV maintains its episomal genome in a tightly condensed chromatin state, with the lytic switch genes BZLF1 and BRLF1 transcriptionally silenced. HDAC inhibition promotes histone hyperacetylation at these gene promoters, leading to chromatin relaxation and derepression of BZLF1 and BRLF1 expression. The encoded proteins then initiate a cascade of viral lytic gene expression, driving the switch from latent to lytic replication (38, 39). This epigenetic reactivation can precipitate life-threatening complications in susceptible patients, particularly those with ENKTL (35, 38).

Although studies suggest that combining HDAC inhibitors with other agents may yield synergistic effects to suppress romidepsin-induced EBV reactivation, clinical evidence supporting this approach remains limited. For instance, a phase II trial of panobinostat combined with bortezomib reported one partial response and one instance of stable disease among R/R ENKTL patients, with no observed EBV reactivation (40). Preclinical findings indicate that phosphodiesterase 5 inhibitors, such as sildenafil, demonstrated efficacy in inhibiting romidepsin-induced EBV reactivation *in vitro*, with a favorable toxicity profile (38). However, these findings lack clinical validation: The PDE5 inhibitor data remains preclinical, and no evidence exists regarding EBV reactivation risk with bortezomib-romidepsin combinations. Consequently, romidepsin remains contraindicated in ENKTL patients due to potentially fatal EBV-driven complications. Heightened vigilance is warranted when administering romidepsin for other lymphomas, particularly EBV-positive lymphoproliferative malignancies.

Cardiac toxicity is a well-established AE associated with romidepsin. Clinical trial data predominantly document asymptomatic arrhythmias, T-wave/ST-segment abnormalities, and corrected QT prolongation (7, 9, 10, 41, 42). A pooled analysis of romidepsin studies in PTCL reported Electrocardiogram (ECG) T-wave changes in 12% (30/250) of patients and tachycardia in 2.4% (6/250), with all events being Grade 1–2 severity (32). Notably, combination regimens, including CHOP (15), platinum/etoposide combinations (43), bendamustine (44), gemcitabine-based protocols (45), anthracyclines (46), and azacitidine (47), demonstrate cardiac safety profiles comparable to chemotherapy monotherapies, with the notable exception of the Ro-CHOP Phase III trial (NCT01796002). During early trials, seven sudden deaths occurred in patients receiving romidepsin. However, all affected individuals had serious pre-existing heart conditions (e.g., valve disease, severe atherosclerosis, prior heart attacks, uncontrolled diabetes/hypertension) (7, 10, 48–52), making it impossible to confirm romidepsin as the cause. This safety profile led to revised eligibility criteria excluding patients with cardiovascular comorbidities or sudden death risks, alongside instituted ECG surveillance. Under these amended protocols, clinical development programs observed no subsequent occurrences of sudden death, sustained ventricular tachycardia, or torsade de pointes. Notably, available evidence does not support a causal association between romidepsin and direct myocardial injury or functional impairment (50, 53–55). Our analysis identified both established and novel cardiac signals. Labeled AEs included electrocardiogram QT prolonged (n = 62, ROR 31.70), electrocardiogram abnormal (n = 6, ROR 16.35). By contrast, previous clinical trials primarily reported QT prolongation as the predominant ECG abnormality, with limited documentation of broader ECG abnormalities. This discrepancy may be attributed to the larger sample size and greater real-world heterogeneity of our analysis, which enabled us to detect less frequent, yet still clinically relevant, ECG changes. The potential new signals identified in our study were cardiac failure acute (n = 4, ROR 10.71), atrial fibrillation (n = 56, ROR 10.99), sinus tachycardia (n = 7, ROR 10.84), mitral valve incompetence (n = 3, ROR 6.85), and bundle branch block left (n = 3, ROR 16.24). Although atrial fibrillation has been mechanistically associated with HDAC inhibitors (56), it was not prominently captured in earlier romidepsin trials, likely due to the relatively small sample sizes, shorter follow-up periods, and stringent enrollment criteria that excluded patients with significant cardiovascular comorbidities. Similarly, acute cardiac failure, mitral regurgitation, and left bundle branch block are novel findings that have not been previously reported in clinical trials. These signals may reflect the greater comorbidity burden and broader patient populations encountered in real-world practice compared with the controlled environments of prospective trials. From a mechanistic perspective, the potential pathophysiological basis for romidepsin-induced acute cardiac failure, and by extension, its proarrhythmic effects, may involve HDAC inhibition-mediated disruption of cardiomyocyte mitochondrial function and calcium handling, as well as altered expression of contractile proteins and ion channels critical for cardiac contractility (57–59). Additionally, HDAC inhibitors have been shown to modulate autophagy and apoptosis pathways in cardiac tissue. Under conditions of stress or pre-existing cardiac compromise, this could lead to acute decompensation and electrical instability (59, 60). Noonan et al. established that romidepsin-induced modulation of cardiac ATP-sensitive K^+^(KATP) channels drives ST-segment depression and ECG abnormalities (51), necessitating strict potassium-focused electrolyte management during therapy. Given the routine co-administration of antiemetics with romidepsin, preferential selection of 5-hydroxytryptamine 3 (5-HT3) receptor antagonists with lower QT prolongation risk (e.g., granisetron (61), palonosetron (62, 63)) over higher-risk agents like ondansetron is advised, alongside vigilant QT interval monitoring and electrolyte maintenance (52). Though routine ECG monitoring is not FDA-mandated, heightened vigilance remains essential for patients with cardiac comorbidities (particularly congenital long QT syndrome). Emerging evidence additionally warrants vigilance for potential novel AEs identified in our analysis during romidepsin therapy. Given the higher prevalence of cardiovascular risk factors and reduced cardiac reserve in older adults, we recommend more intensive cardiovascular monitoring in this population, paying particular attention to individuals with pre-existing cardiovascular conditions or multiple risk factors. This includes baseline and periodic ECG assessments (e.g., prior to each cycle or at least every two cycles) and regular cardiac function evaluations (e.g., baseline echocardiography and as clinically indicated) to facilitate the early detection and timely intervention of acute cardiac failure, arrhythmias, and other newly identified cardiac signals (55, 64).

As noted above, romidepsin is often used alongside various chemotherapeutic agents, including CHOP, platinum/etoposide, bendamustine, gemcitabine-based regimens, anthracyclines, and azacitidine. The phase III Ro-CHOP trial (NCT01796002) compared romidepsin plus CHOP versus CHOP alone in patients with previously untreated PTCL, and reported comparable overall safety profiles between the two groups. However, a higher incidence of hematologic toxicities (thrombocytopenia: 50% vs 10%, neutropenia: 49% vs 33%, anemia: 47% vs 17%) and infections was observed in the combination group (11). Studies have shown that romidepsin combined with platinum/etoposide has a manageable safety profile, with myelosuppression being the most common toxicity and no significant increase in cardiac or hepatic events compared with chemotherapy alone (43). Similarly, romidepsin in combination with bendamustine demonstrated acceptable tolerability with minimal toxicity. Hematological toxicity was mild and lower than with other romidepsin-based combinations, while significant nausea and vomiting occurred in over 50% of patients (44). A multicenter phase 2 study of romidepsin combined with oral azacitidine reported the most frequent grade 3 to 4 AEs as thrombocytopenia (48%), neutropenia (40%), lymphopenia (32%), and anemia (16%) (65). However, direct comparisons of AE profiles across different combination regimens remain limited, as most available data derive from phase I/II trials with small sample sizes and heterogeneous patient populations. It is notable that the safety profile of romidepsin appears to be influenced by the specific combination partner, with certain regimens potentially amplifying the risk of specific toxicities such as myelosuppression or infection. These observations underscore the importance of individualized treatment selection and vigilant monitoring based on the specific combination regimen employed.

The romidepsin RCP provides established algorithms for modifying the dose in response to specific AEs. For hematologic toxicities, grade 3 or 4 thrombocytopenia or neutropenia require treatment interruption until platelet counts recover to ≥ 75 × 10^9^/L and ANC to ≥ 1.0 × 10^9^/L, after which treatment can resume at the same dose. Recurrence warrants a reduction in dosage, and prolonged cytopenias or ongoing reductions in dosage may require permanent discontinuation (66). For non-hematologic toxicities (excluding alopecia, nausea, and vomiting), grade 3 or 4 events require interruption until resolution to grade ≤ 1 or baseline, with a dose reduction upon recurrence (66). For hepatic impairment, dose adjustments are based on bilirubin levels: no adjustment for mild impairment; 7 mg/m² for moderate impairment (bilirubin 1.5-3× ULN), and 5 mg/m² for severe impairment (bilirubin > 3× ULN) (66). When these algorithms are compared with our real-world findings, several observations emerge. First, the RCP algorithms address thrombocytopenia and neutropenia through specific dose modification thresholds, all of which were confirmed as significant signals in our study, thus supporting continued current practices. Second, our analysis identified novel signals, including acute cardiac failure, atrial fibrillation, hepatic failure, and decreased glomerular filtration rate, which are not explicitly covered in the RCP guidance. Third, while the RCP states that no dose adjustment is required for renal impairment when CrCl > 15 mL/min, our finding of a decreased glomerular filtration rate suggests that renal function may warrant closer monitoring. These findings have implications for clinical practice and potential RCP updates. The detection of serious events that are not currently addressed by the RCP algorithms suggests that the current guidance on dose modifications may need to be expanded to encompass a broader spectrum of real-world toxicities. The absence of specific algorithms for these newly identified signals underscores the need for greater vigilance and individualized management, particularly in patients with baseline comorbidities.

In addition to the newly identified cardiotoxicities, this study also revealed several other significant potential AEs associated with romidepsin, including hepatic failure, amenorrhea, mental status changes, glomerular filtration rate decreased, embolism, and retinal detachment. These events extend beyond those currently listed in FDA documentation. Their detection may have been challenging in smaller or more rigidly controlled clinical trials, underscoring the need for ongoing safety surveillance and comprehensive pharmacovigilance of romidepsin.

While no direct studies or reports have established a link between romidepsin and embolism, other HDAC inhibitors, such as vorinostat, have demonstrated this association. In a phase IIb multicenter trial of vorinostat for patients with persistent, progressive, or refractory CTCLs, embolism occurred in 5.4% (4/74) of patients (67). In contrast, embolism has not been widely reported in romidepsin clinical trials, which may be attributed to the relatively small sample sizes, shorter treatment durations, and exclusion of patients with significant thromboembolic risk factors in those studies. The detection of this signal in our analysis is probably due to the inclusion of a more heterogeneous, real-world population with a higher baseline burden of comorbidities. Regarding hepatic safety, clinical trials of romidepsin for CTCL and PTCL patients reported transient and mild elevations in serum enzymes during treatment in 7-20% of cases, but these abnormalities were typically self-limiting and mild, not requiring dose adjustment, and no instances of clinically apparent liver injury were documented (7, 10, 68). However, our study detected a strong positive pharmacovigilance signal for hepatic failure associated with romidepsin (ROR 7.83), consistent with prior findings (56). This apparent contradiction between clinical trial data and real-world signals may be explained by differences in patient selection. Trials often exclude individuals with baseline hepatic impairment or significant comorbidities (69), whereas real-world settings include such vulnerable populations. Furthermore, the use of longer treatment durations and concomitant medications in clinical practice may lead to severe hepatic events that are not captured in controlled trial settings. Based on these findings, we recommend a baseline assessment of liver function prior to treatment initiation, followed by regular monitoring at least every two cycles. More frequent evaluation is required for patients with baseline abnormalities, pre-existing hepatic impairment, or who are taking potentially hepatotoxic medications (68, 70). For other identified signals (including amenorrhea, mental status changes, glomerular filtration rate decreased, and retinal detachment), comparative data from romidepsin clinical trials are limited, most likely due to insufficient sample sizes, short follow-up periods, and the fact that trials primarily focus on common rather than rare AEs. Identifying these signals in our study highlights the value of using large-scale, real-world pharmacovigilance data alongside evidence from prospective clinical trials. These newly identified potential AEs associated with romidepsin may be unexpected and warrant further exploration. Although their clinical significance and underlying mechanisms remain incompletely understood, these findings necessitate vigilant monitoring of an expanded profile of potential AEs during romidepsin therapy. In particular, given the newly identified signal of decreased glomerular filtration rate, we recommend obtaining a baseline serum creatinine level and estimated glomerular filtration rate (eGFR) prior to initiating treatment, with periodic reassessment (e.g., at least every two cycles) to enable the early detection of potential renal impairment (56). The possible pathophysiological pathways underlying romidepsin-induced retinal detachment merit in-depth investigation among these signals. As an HDAC inhibitor, romidepsin may disrupt retinal homeostasis by altering the acetylation levels of structural proteins, transcription factors or inflammatory mediators. This could potentially compromise the integrity of the retinal pigment epithelium and the neurosensory retina (71, 72). Elucidating such mechanisms will be critical for informing risk assessment and clinical management.

Utilizing the FAERS database for large-scale, real-world population studies offers significant advantages, primarily the ability to analyze vast numbers of exposure reports and investigate rare AEs. First, as a spontaneous reporting system, FAERS is prone to misreporting, under-reporting, duplicates, and incomplete data. In this study, we encountered substantial missing data for weight (62.88%), concomitant medications (33.15%), and time-to-onset (78.52%), which may introduce bias and compromise the accuracy and completeness of our findings. Second, the FAERS database inherently contains reports from diverse geographic regions and varying levels of reporter expertise (healthcare professionals vs. consumers), leading to inevitable data quality challenges. Fortunately, in this study, 95.77% of reports originated from health professionals, significantly enhancing the data’s overall credibility. Third, the database does not capture the total number of exposed patients, precluding AE incidence rate calculation. Fourth, the absence of CTCAE-based severity grading (where events are classified as either “serious” or “non-serious”) meant that a stratified analysis by severity level could not be performed. To mitigate these limitations and guide future research, several strategies could be adopted. These include applying multiple imputation techniques to address missing continuous variables (73), integrating multi-database triangulation (e.g., VigiBase and electronic health records) to cross-validate findings and compensate for under-reporting (74), and implementing duplicate detection algorithms to improve data quality (25). Additionally, future studies using electronic health records could facilitate severity-stratified analyses and incidence rate calculations. This would overcome the key limitations of spontaneous reporting systems (75). Despite these limitations, FAERS remains an indispensable tool for post-marketing pharmacovigilance, providing critical insights into drug safety trends. This study systematically characterized romidepsin-associated AE signals, identifying clinically relevant risks. These findings contribute to an enhanced understanding of romidepsin’s safety profile and its clinical application, though they should be interpreted with caution, considering the study’s methodological constraints.

## 5 Conclusion

This comprehensive pharmacovigilance analysis of FAERS data identified key safety signals associated with romidepsin, confirming known risks documented in the RCP, such as thrombocytopenia, pyrexia, anemia, QT interval prolongation, and infections, while also detecting novel signals not currently described in the label. These include cardiotoxicities beyond QT prolongation, namely acute cardiac failure, atrial fibrillation, sinus tachycardia, mitral valve incompetence, and bundle branch block left, as well as other significant AEs, including hepatic failure, amenorrhea, mental status changes, glomerular filtration rate decreased, embolism, and retinal detachment. These real-world pharmacovigilance findings have important translational value. Identifying previously undocumented AEs supports updates to the romidepsin prescribing information and provides valuable insights for regulatory risk communication. Furthermore, the monitoring recommendations derived from this study, such as routine cardiac, hepatic, and renal function assessments, offer a practical framework for refining clinical practice guidelines. Although requiring cautious interpretation due to database-related constraints, these findings provide valuable real-world insights into romidepsin’s evolving safety profile.

## Data Availability

The original contributions presented in the study are included in the article/**Supplementary Material**. Further inquiries can be directed to the corresponding authors.
