# Supplementary material for: A real-world pharmacovigilance study of romidepsin based on FDA adverse event reporting system database
Source: Front Oncol. 2026 Jun 10;16:1694602. doi: 10.3389/fonc.2026.1694602 (PMC13290537; doi:10.3389/fonc.2026.1694602)
Supplement: Supplementary file 1 [file Table1.docx]

**Supplementary materials**

**Table S1** Two-by-two contingency (2×2) table for disproportionality analyses.

|  | **Target adverse event** | **All other adverse events** | **Total** |
| --- | --- | --- | --- |
| **Target drug** | a | b | a+b |
| **All other drugs** | c | d | c+d |
| **total** | a+c | b+d | a+b+c+d |

**Equation:** a, number of reports containing both the target drug and target adverse drug reaction; b, number of reports containing other adverse drug reaction of the target drug; c, number of reports containing the target adverse drug reaction of other drugs; d, number of reports containing other drugs and other adverse drug reactions.

**Table S2** algorithms used for signal detection.

| **Algorithms** | **Equation** | **Criteria** |
| --- | --- | --- |
| ROR | ROR = ad/c/b | ROR05 > 1, n ≥ 2 |
|  | 95%CI = e^ln(ROR)±1.96(1/a+1/b+1/c+1/d)^0.5^ |  |
| PRR | PRR = [a/(a+b)]/[c/(c + d)] | PRR ≥ 2 |
|  | χ^2^ = [(ad-bc)^2^ (a+b + c + d)]/[(a+b)(c + d)(a+c)(b + d)] | χ^2^ ≥ 4, n ≥ 3 |
| BCPNN | IC = log2 [a (a+b + c + d)]/[(a+c)(a+b)] | IC025 > 0 |
|  | 95%CI = e^ln(IC)±1.96(1/a+1/b+1/c+1/d)^0.5^ |  |
| MGPS | EBGM = a (a+b + c + d)/(a+c)/(a+b) | EBGM05 > 2, N ≥ 0 |
|  | 95%CI = e^ln(EBGM)±1.96(1/a+1/b+1/c+1/d)^0.5^ |  |

**Abbreviations:** N, number of adverse event reports; CI, confidence interval; ROR, reporting odds ratio; ROR05, the lower limit of the 95 two-sided CI of the ROR; N, the number of co-occurrences; PRR, proportional reporting ratio; χ^2^, chi-squared; BCPNN, bayesian confidence propagation neural network; IC, information component; IC025, the lower limit of the 95 two-sided CI of the IC; MGPS, multi-item gamma Poisson shrinker; EBGM, empirical bayesian geometric mean; EBGM05, the lower 95 two-sided CI of EBGM.

**Table S3** The signal strength of reports of romidepsin at PTs level in the FAERS database (n ≥ 3).

(Signals that may be attributed to disease progression or other non-pharmaceutical factors)

| **Preferred Terms (PTs)** | **Number** | **ROR (95% CI)** | **PRR (χ^2^)** | **IC (IC025)** | **EBGM**  **(EBGM05)** |
| --- | --- | --- | --- | --- | --- |
| Peripheral T-cell lymphoma unspecified | 106 | 4472.49 (3578.96-5589.09) | 4334.18 (343041.53) | 11.66 (9.33) | 3237.96 (2591.07) |
| Disease progression | 45 | 6.89 (5.13-9.24) | 6.81 (223.35) | 2.77 (2.06) | 6.81 (5.07) |
| Malignant neoplasm progression | 44 | 7.28 (5.41-9.80) | 7.20 (235.09) | 2.85 (2.11) | 7.19 (5.34) |
| Product storage error | 44 | 6.74 (5.00-9.07) | 6.66 (212.05) | 2.74 (2.03) | 6.66 (4.95) |
| Angioimmunoblastic T-cell lymphoma | 37 | 2559.87 (1796.23-3648.15) | 2532.24 (78155.55) | 11.05 (7.75) | 2114.14 (1483.47) |
| Cutaneous T-cell lymphoma | 19 | 182.06 (115.62-286.70) | 181.06 (3354.89) | 7.48 (4.75) | 178.55 (113.39) |
| Therapy partial responder | 17 | 19.59 (12.16-31.56) | 19.50 (297.97) | 4.28 (2.66) | 19.47 (12.09) |
| T-cell lymphoma | 16 | 315.05 (191.64-517.92) | 313.58 (4866.22) | 8.26 (5.02) | 306.11 (186.20) |
| Acute myeloid leukaemia | 13 | 16.39 (9.50-28.26) | 16.33 (186.86) | 4.03 (2.34) | 16.31 (9.46) |
| Haemophagocytic lymphohistiocytosis | 11 | 19.06 (10.54-34.47) | 19.00 (187.37) | 4.25 (2.35) | 18.98 (10.49) |
| Lymphoma | 8 | 9.49 (4.74-19.00) | 9.47 (60.60) | 3.24 (1.62) | 9.47 (4.73) |
| Disseminated intravascular coagulation | 7 | 11.72 (5.58-24.62) | 11.70 (68.45) | 3.55 (1.69) | 11.69 (5.57) |
| Anaplastic large-cell lymphoma | 7 | 398.98 (187.90-847.16) | 398.16 (2689.50) | 8.59 (4.05) | 386.18 (181.87) |
| Graft versus host disease | 6 | 17.45 (7.83-38.89) | 17.42 (92.75) | 4.12 (1.85) | 17.40 (7.81) |
| Diffuse large B-cell lymphoma | 5 | 11.14 (4.63-26.79) | 11.12 (46.03) | 3.47 (1.44) | 11.11 (4.62) |
| Myelodysplastic syndrome | 5 | 6.95 (2.89-16.70) | 6.94 (25.40) | 2.79 (1.16) | 6.93 (2.88) |
| Non-Hodgkin's lymphoma | 4 | 13.20 (4.95-35.22) | 13.19 (45.02) | 3.72 (1.39) | 13.18 (4.94) |
| Adult T-cell lymphoma/leukaemia | 4 | 141.97 (52.97-380.55) | 141.81 (553.13) | 7.13 (2.66) | 140.26 (52.33) |
| Metastases to central nervous system | 4 | 5.68 (2.13-15.14) | 5.67 (15.39) | 2.50 (0.94) | 5.67 (2.13) |
| Acute graft versus host disease | 4 | 17.90 (6.71-47.75) | 17.88 (63.66) | 4.16 (1.56) | 17.86 (6.69) |
| Graft versus host disease in gastrointestinal tract | 4 | 26.65 (9.99-71.12) | 26.62 (98.43) | 4.73 (1.77) | 26.57 (9.96) |
| Chronic graft versus host disease | 3 | 16.04 (5.17-49.81) | 16.03 (42.23) | 4.00 (1.29) | 16.01 (5.16) |
| Invasive ductal breast carcinoma | 3 | 17.05 (5.49-52.93) | 17.03 (45.22) | 4.09 (1.32) | 17.01 (5.48) |
| Malignant pleural effusion | 3 | 34.46 (11.09-107.07) | 34.43 (97.13) | 5.10 (1.64) | 34.34 (11.05) |
| Peripheral T-cell lymphoma unspecified stage IV | 3 | 1372.42 (417.04-4516.50) | 1371.22 (3710.16) | 10.27 (3.12) | 1238.62 (376.38) |
| Squamous cell carcinoma of skin | 3 | 8.60 (2.77-26.68) | 8.59 (20.11) | 3.10 (1.00) | 8.59 (2.77) |
| Peripheral T-cell lymphoma unspecified refractory | 3 | 4269.77 (1155.42-15778.61) | 4266.03 (9594.07) | 11.64 (3.15) | 3199.77 (865.87) |
| Cutaneous T-cell lymphoma recurrent | 3 | 512.37 (161.51-1625.47) | 511.92 (1470.94) | 8.94 (2.82) | 492.27 (155.17) |
| Graft versus host disease in skin | 3 | 20.92 (6.74-64.95) | 20.90 (56.75) | 4.38 (1.41) | 20.87 (6.72) |
| Post transplant lymphoproliferative disorder | 3 | 9.52 (3.07-29.53) | 9.51 (22.83) | 3.25 (1.05) | 9.50 (3.06) |

**Abbreviations:** FAERS, FDA Adverse Event Reporting System; ROR, reporting odds ratio; CI, confidence interval; PRR, proportional reporting ratio; χ^2^, chi-squared; IC, information component; EBGM, empirical Bayesian geometric mean.

**Table S4** Top 10 PTs by case count for romidepsin in the FAERS database (n ≥ 3).

| **Preferred Terms (PTs)** | **Number** | **ROR (95% CI)** | **PRR (χ^2^)** | **IC (IC025)** | **EBGM**  **(EBGM05)** |
| --- | --- | --- | --- | --- | --- |
| Thrombocytopenia | 90 | 16.00 (12.98-19.73) | 15.61 (1231.29) | 3.96 (3.21) | 15.59 (12.65) |
| Pyrexia | 80 | 4.48 (3.59-5.59) | 4.40 (210.93) | 2.14 (1.71) | 4.39 (3.52) |
| Anaemia | 63 | 6.44 (5.02-8.26) | 6.34 (283.96) | 2.66 (2.08) | 6.34 (4.94) |
| Electrocardiogram QT prolonged | 62 | 31.70 (24.65-40.77) | 31.15 (1805.87) | 4.96 (3.86) | 31.08 (24.17) |
| Platelet count decreased | 61 | 10.62 (8.24-13.68) | 10.45 (521.66) | 3.38 (2.63) | 10.44 (8.10) |
| Atrial fibrillation | 56 | 10.99 (8.44-14.31) | 10.83 (499.84) | 3.44 (2.64) | 10.82 (8.31) |
| Neutropenia | 50 | 6.52 (4.93-8.62) | 6.44 (230.10) | 2.69 (2.03) | 6.44 (4.87) |
| Decreased appetite | 41 | 3.13 (2.30-4.26) | 3.10 (58.70) | 1.63 (1.20) | 3.10 (2.28) |
| Febrile neutropenia | 38 | 10.56 (7.67-14.54) | 10.45 (324.87) | 3.38 (2.46) | 10.44 (7.58) |
| Sepsis | 29 | 4.97 (3.45-7.16) | 4.93 (91.05) | 2.30 (1.60) | 4.93 (3.42) |

**Abbreviations:** FAERS, FDA Adverse Event Reporting System; ROR, reporting odds ratio; CI, confidence interval; PRR, proportional reporting ratio; χ^2^, chi-squared; IC, information component; EBGM, empirical Bayesian geometric mean.

**Table S5** Top 10 PTs by ROR for romidepsin in the FAERS database (n ≥ 3).

| **Preferred Terms (PTs)** | **Number** | **ROR (95% CI)** | **PRR (χ^2^)** | **IC (IC025)** | **EBGM**  **(EBGM05)** | |
| --- | --- | --- | --- | --- | --- | --- |
| Epstein-Barr virus infection reactivation | 6 | 80.46 (36.03-179.68) | 80.32 (467.09) | 6.32 (2.83) | 79.83 (35.75) |  |
| Tumour lysis syndrome | 20 | 41.02 (26.41-63.70) | 40.78 (773.82) | 5.35 (3.44) | 40.66 (26.18) |  |
| Cytomegalovirus chorioretinitis | 4 | 37.27 (13.96-99.51) | 37.23 (140.63) | 5.21 (1.95) | 37.13 (13.91) |  |
| Electrocardiogram QT prolonged | 62 | 31.70 (24.65-40.77) | 31.15 (1805.87) | 4.96 (3.86) | 31.08 (24.17) |  |
| Pneumonia klebsiella | 3 | 30.33 (9.76-94.21) | 30.30 (84.81) | 4.92 (1.58) | 30.23 (9.73) |  |
| Cytomegalovirus viraemia | 6 | 23.87 (10.71-53.22) | 23.83 (131.01) | 4.57 (2.05) | 23.79 (10.67) |  |
| Cytomegalovirus infection reactivation | 6 | 23.59 (10.58-52.59) | 23.55 (129.35) | 4.56 (2.04) | 23.51 (10.55) |  |
| Febrile bone marrow aplasia | 5 | 21.86 (9.08-52.58) | 21.82 (99.19) | 4.45 (1.85) | 21.79 (9.06) |  |
| Epstein-Barr virus infection | 7 | 20.85 (9.93-43.80) | 20.81 (131.80) | 4.38 (2.08) | 20.78 (9.89) |  |
| Cytopenia | 16 | 20.65 (12.63-33.75) | 20.56 (297.25) | 4.36 (2.67) | 20.52 (12.55) |  |

**Abbreviations:** FAERS, FDA Adverse Event Reporting System; ROR, reporting odds ratio; CI, confidence interval; PRR, proportional reporting ratio; χ^2^, chi-squared; IC, information component; EBGM, empirical Bayesian geometric mean.
